# Periodic breathing in patients with stable obstructive sleep apnea on long-term continuous positive airway pressure treatment: a retrospective study using CPAP remote monitoring data

**DOI:** 10.1007/s11325-021-02510-0

**Published:** 2021-10-14

**Authors:** Kimimasa Saito, Yoko Takamatsu

**Affiliations:** Saito Naika Kokyukika, Mie Sleep Clinic, Ise-shi, 519-0502 Japan

**Keywords:** Cheyne-Stokes breathing, Periodic breathing, CPAP remote monitoring, Atrial fibrillation, QRS duration, Obstructive sleep apnea

## Abstract

**Purpose:**

The purpose of this study was to investigate the rate of periodic breathing (PB) and factors associated with the emergence or persistence of PB in patients with obstructive sleep apnea (OSA) by continuous positive airway pressure (CPAP) remote monitoring data.

**Methods:**

This was a retrospective cohort study on 775 patients who had used the same model CPAP machine for at least 1 year as of September 1, 2020. The data were analyzed online using the dedicated analysis system. Using exporter software, average apnea/hypopnea index (AHI), average central apnea index (CAI), and average the rate of PB time (PB%) were cited.

**Results:**

Among 618 patients analyzed (age 61.7 ± 12.2 years, male 89%, BMI 27.2 ± 4.9), the average duration of CPAP use was 7.5 ± 4.0 years. The median PB% in stable patients was low at 0.32%, and only 149 patients (24%) had a PB% above 1%. Multiple regression analysis of factors for the development of PB showed that the most important factor was atrial fibrillation (Af) with a coefficient of 0.693 (95% CI; 0.536 to 0.851), followed by QRS duration with a coefficient of 0.445 (95% CI; 0.304 to 0.586), followed by history of heart failure, male sex, comorbid hypertension, obesity, and age. The average PB% for paroxysmal Af was significantly lower than that for persistent and permanent Af.

**Conclusions:**

The median PB% in stable patients on CPAP treatment was low at 0.32%, with only 24% of patients having PB% ≥ 1%. Persistent Af and an increase in QRS duration were found to be important predictors of increased PB%.

**Clinical trial registration:**

UMIN000042555 2021/01/01.

**Supplementary Information:**

The online version contains supplementary material available at 10.1007/s11325-021-02510-0.

## Introduction

The current International Classification of Sleep Disorders (ICSD-3) classifies central sleep apnea (CSA) into 8 types based on the presence or absence of Cheyne-Stokes breathing (CSB) and other criteria [1]. However, the pathophysiological significance of CSA-CSB is still controversial.

Important predisposing factors of CSA-CSB include congestive heart failure (CHF), cerebrovascular disorder, and, probably, renal failure. In clinical practice, we often experience cases in which the frequency of CSA-CSB increases with exacerbation of left ventricular systolic heart failure and decreases or disappears with improvement of heart failure.

In other words, the occurrence and frequency of CSA-CSB may vary, depending on the disease conditions.

Since publication of the results of the SAVE-HF (Treatment of Predominant Central Sleep Apnea by Adaptive Servo-ventilation in Patients with Heart Failure) trial [2], a study demonstrating that adaptive servo-ventilation (ASV) does not alleviate nocturnal cardiovascular stress in patients with systolic heart failure and predominant CSA [3], the importance of CSA-CSB as a treatment target, particularly in patients with impaired left ventricular function, has been reduced. On the other hand, another study demonstrated that a reduction of pulmonary congestion as achieved by a decrease of left atrial pressure through successful MitraClip implantation is associated with the improvement of CSA-CSB [4].

In real-world clinical practice, we often experience that remote monitoring of periodic breathing (PB) may allow much earlier detection of new onset and exacerbation of pathological conditions, especially cardiovascular disease, in patients with OSA [5–7].

The role of CSA-CSB or PB as a prognostic marker rather than a therapeutic target in all patients per se is increasing.

The current study focused on PB in patients with stable obstructive sleep apnea (OSA) undergoing treatment with continuous positive airway pressure (CPAP) for long term.

With the final goal of predicting the onset or exacerbation of cardiovascular events by remote monitoring of PB% in patients with OSA on CPAP, we retrospectively analyzed CPAP remote monitoring data to determine PB% and identify factors associated with the emergence or persistence of PB in patients with stable OSA on CPAP.

## Methods

### Participants

We conducted a retrospective cohort study on 775 patients who had used the Dream Station Auto CPAP machine (Philips Respironics, Murrysville, PA) for at least 1 year at Mie Sleep Clinic as of September 1, 2020. All patients were diagnosed as having OSA, with an apnea–hypopnea index (AHI) ≥ 20 by overnight PSG, or having sleep apnea syndrome (SAS), predominantly of obstructive type, with AHI ≥ 40 by out-of-center sleep testing (OCST), and have been continuously treated with CPAP.

Exclusion criteria were as follows: age ≥ 90 years as of September 1, 2020; poorly adherent patients with a percentage of days of CPAP use of < 50% or an average CPAP use time of < 4 h between September 1 and 30, 2020; and new onset of heart failure, worsening of chronic heart failure, or new onset or relapse of atrial fibrillation (Af) or other cardiovascular diseases in the 3 months before or after September 1, 2020 (i.e., between June 1, 2020, and November 30, 2020).

This study was approved by the ethics committee of the Medical Corporation MSC (#20,002). Written consent was obtained from all patients. This study is registered in the UMIN Clinical Trials Registry (UMIN000042555).

The primary endpoint was the calculation of standard data, such as the monthly median PB%, and the analysis of factors associated with PB in patients with stable OSA on CPAP. The secondary endpoint was subgroup analyses of factors that were strongly associated with PB.

### Clinical data collection

The following baseline characteristic data were extracted from the Medical Corporation MSC Mie Sleep Clinic database, which contained data from October 1, 2007, to November 30, 2020, with the base point set at September 1, 2020 (Table 1):

- Age, sex, BMI
- Average number of years of CPAP use
- Smoking status (former smoker, current smoker, or nonsmoker)
- Frequency of alcohol consumption (daily, ≥ 3/week, 1–2/week, < 1/week, rarely, or never)
- QRS width on ECG within 1 year

**Table 1:** Baseline characteristics

Variables	OSA (n = 618)
Male (%)	549 (89)
Age, year	61.7 ± 12.2
BMI, kg/m^2^	27. 2 ± 4.9
Usage years of CPAP, year	7.5 ± 4.0
Smoking status, n (%)	
Current smoker	99 (16)
Former smoker	269 (44)
Nonsmoker	250 (41)
Frequency of alcohol consumption, n (%)	
≥ 3 times/wk	148 (24)
1–2 times/wk	97 (16)
< 1 times/wk	58 (9)
Rarely or nevever	315 (51)
History of congestive heart failure	29 (5)
History of ischemic heart disease	45 (7)
Prior stroke/TIA	31 (5)
Chronic kidney disease above stage3b	28 (5)
eGFR(Cr), mL/min/1.73m^2^	71.0 ± 16.0
Artrial fibrillation	60 (10)
Hypertension	300 (49)
Type 2 diabetes	112 (18)
Dyslipidemia	206 (33)
Chronic obstructive pulmonary disease	156 (25)
Pacemaker implanted	7 (1)
QRS duration on ECG,ms	89.7 ± 13.8
Central nervous system agents	45 (7)

Data are presented as n (%) or average ± SD

*BMI,* body mass index; *CPAP,* continuous positive airway pressure; *TIA,* transient ischemic attack; *ECG,* electrocardiogram

Data on comorbidities and patients’ medication status were obtained from the database or from the medical records of patients following physician referral. The diagnoses of heart failure, ischemic heart disease, and Af were based on diagnoses by cardiologists at neighboring flagship hospitals.

- Comorbidities and past medical history
  History of congestive heart failure, ischemic heart disease, Af, pacemaker (including cardiac resynchronization therapy (CRT) device) implantation, prior stroke/transient ischemic attack (TIA), hypertension, diabetes, dyslipidemia, stage ≥ 3b CKD, and chronic obstructive pulmonary disease.
- Medications

Table 2 shows the further classification of Af type (paroxysmal, persistent, or permanent), QRS complex, and central nervous system (CNS) agents. We included four medication classes as CNS agents: opioids, benzodiazepines, sedatives, and non-benzodiazepine agonists.

**Table 2:** Sleep variables detected by CPAP remote monitoring during 1 month (n = 618)

Variables	
CPAP usage time, min	381.0 ± 74.5
CPAP usage day, %	95.5 ± 9.4
AHI, no/hr	3.1 (0.2–36.6)
Log AHI	0.48 ± 0.31
CAI,no/hr	0.12 (0–15.6)
Log CAI	-0.83 ± 0.48
PB%Device,%	0.32 (0–32.8)
75th percentile PB,%	0.94
Avbove 1%	149 (24.1)
Log PB%Device	-0.43 ± 0.65
Large leak,%	0.52 (0–55.3)
Log Large leak	-0.03 ± 0.82

Data are presented as average ± SD, median (range),75th percentile or n (%)

*CPAP,* continuous positive airway pressure; *AHI,* apnea/hypopnea index;

*CAI,* clear airway (central apnea) index; *PB,* periodic breathing

### Polysomnography

Full attended overnight polysomnography (PSG) was conducted in a laboratory setting, which was classified as type 1 sleep study by the American Academy of Sleep Medicine (AASM). PSG was performed using the Alice® 4 or Alice® 6 system (Philips Respironics, Murrysville, PA). Sleep and respiratory event scoring was carried out according to the AASM manual. Our clinic is specialized in pulmonary medicine. All patients with a history of heart failure underwent diagnostic PSG in the stable compensatory phase of heart failure.

### Definitions of PB% detected during diagnostic PSG and by the CPAP device

The AASM recommends scoring a respiratory event as Cheyne-Stokes breathing if both of the following criteria are met:

- There are episodes of at least three consecutive central apneas and/or central hypopneas separated by a crescendo and decrescendo change in breathing amplitude, with a cycle length of at least 40 s (typically 45 to 90 s).
- There are five or more central apneas and/or central hypopneas per hour associated with the crescendo/decrescendo breathing pattern recorded over a minimum of 2 h of monitoring.

According to the algorithm of the CPAP device, the device detects PB that meets only criterion a. Thus, in this study, we defined PB as follows:

- **PB%PSG**, defined as the percentage (%) of the time exhibiting PB to total sleep time (TST) during diagnostic PSG
- **PB%Device**, defined as the percentage (%) of the time exhibiting PB to the duration of the CPAP device use recorded in the CPAP data

Patients with PB%Device ≥ 2% were divided into the emergence group that included those with PB%PSG < 1% and the persistence group that included those with PB%PSG ≥ 1%.

The clinical factors associated with the emergence or persistence of PB were investigated in these groups.

### CPAP titration

At our clinic, CPAP treatment is initiated in the Auto CPAP mode on an outpatient basis (Supplemental Figure). When the treatment is initiated, the upper limit of pressure is set at a value estimated to not be excessive, according to the body weight of each patient. Within 1 week of treatment initiation, daily remote monitoring was initiated. The association between pressure variation and respiratory events, as well as leak patterns, is closely monitored, in addition to the AHI calculated using the CPAP device. The patient was re-examined at the outpatient clinic within 2–4 weeks after treatment initiation. If obstructive events persist (AHI ≥ 10, PB%Device < 2%) after the set pressure upper limit was reached, we would raise the pressure limit further. When a patient exhibits PB and a large leak with pressure reaching the upper limit or increasing despite an AHI ≥ 10, PB%Device ≥ 2%, and a decreased incidence of obstructive events, the patient is regarded to have treatment-emergent central sleep apnea (TECSA). The pressure at the occurrence of PB was assumed to be the break point, and the upper limit of the pressure was adjusted to be lower than this point. This adjustment leads to resolution of the TECSA pattern within 3 months in most patients without comorbid heart or cerebrovascular disease. When the TECSA pattern persists for 3 months, patients are admitted for manual CPAP titration under PSG. Meanwhile, patients suspected of having comorbid heart failure or other heart or cerebrovascular diseases, and who provided consent, underwent measurement of brain natriuretic peptide (BNP), electrocardiography, and echocardiography, according to different criteria (AHI15 and CSR > 2%). The patients were then admitted for manual adaptive servo-ventilation (ASV) titration under PSG within 3 to 6 months. Patients indicated for ASV are transitioned to ASV.

CHF patients with TECSA pattern sets the minimum pressure support of the ASV to 0 cmH2O. This study included patients who had been continuously treated with CPAP for 1 year or longer, after undergoing the procedure described above at our clinic.

### Sleep data collection

The CPAP device used was Dream Station Auto. The device periodically records the user’s breathing signals and analyzes the data in near real time. It also records raw respiratory data at random. Analysis results are automatically uploaded to a central database. The data were analyzed online using the dedicated analysis system (EncoreAnywhere, ver. 2.44; Philips Respironics) and cited using the CareExporter software (version 1.11.1.0; Philips Respironics). The data cited were average apnea/hypopnea index (AHI), average central apnea (clear airway) index (CAI), PB%Device, average percentage of large leak on days of CPAP use (LL%), percentage of days of CPAP use (%), and average CPAP use time (on days of use, min). The monitoring data from the CPAP device was averaged over a 30-day period starting on September 1, 2020.

### Statistical analysis

The Kolmogorov–Smirnov test was used to test the normal distribution of continuous variables. Data are presented as average ± SD, median (range), 75th percentile, or *n* (%). The data for AHI, CAI, PB%Device, and LL% showed a pointed distribution, expressed as median (full range), and transformed to 10-based logarithmic scale to obtain a normal distribution. The correlation among log AHI, log CAI, and log PB%Device was analyzed using the Pearson product-moment correlation coefficient. For the analysis of factors associated with PB, multiple regression analysis was used to create a prediction formula for log PB%Device. For explanatory variables, those that were considered to be clinically important based on previous studies were added to the model. In addition to age and sex, smoking history and obesity, which are cardiovascular risk factors, as well as factors previously identified as being associated with PB, including use of CNS agents, such as alcohol and opioid [8, 9], history of congestive heart failure, history of stroke/TIA, history of pacemaker implantation, Af, hypertension, concomitant stage ≥ 3b CKD [10], QRS duration on ECG [11–13], percent large leak calculated from CPAP device data [7, 14], and average CPAP use time [15], were considered to be related to PB onset and analyzed as binary variables, where BMI ≥ 30, CKD stage ≥ 3b, QRS ≥ 110 ms, and LL% ≥ 15% were coded as 1. The difference in average log PB%Device between different Af types was analyzed using one-way analysis of variance (ANOVA), followed by multiple comparison using Tukey’s post hoc test. The difference in QRS duration between the two groups was analyzed using Welch’s *t*-test.

Probability values ≤ 0.05 were considered significant. All statistical analyses were performed with EZR Ver.1.52 (Saitama Medical Center, Jichi Medical University, Saitama, Japan), which is a graphical user interface for the R version 4.02 (The R Foundation for Statistical Computing, Vienna, Austria). Specifically, it is a modified version of the R commander designed to add statistical functions frequently used in biostatistics.

## Results

### Baseline characteristics

**Fig. 1:**
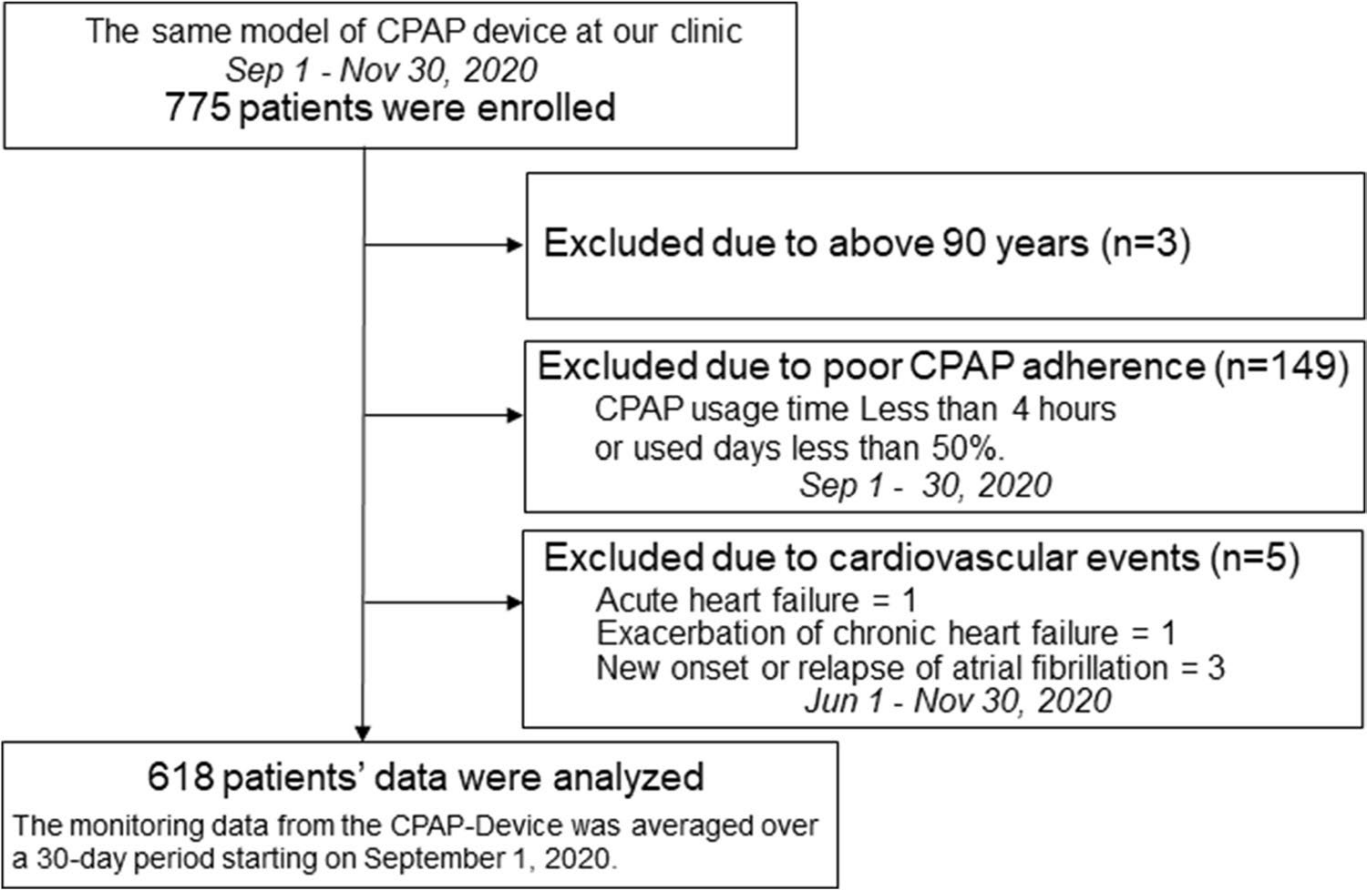
Flow diagram of the study subject selection process. *CPAP*, continuous positive airway pressure

A total of 775 patients were enrolled, of whom 3 were aged ≥ 90 years, 149 were poorly adherent to CPAP treatment, and 5 had cardiovascular events within 3 months before or after analysis. Consequently, 618 patients were considered as having stable OSA and were included in the analysis (Fig. 1). Table 1 lists the baseline characteristics of the analyzable patients. The average duration of CPAP use by the 618 patients was 7.5 ± 4.0 years (1–13 years). The average age was 61.7 ± 12.2 years, men accounted for 88.8% of all patients, and the average BMI was 27.2 ± 4.9 kg/m^2^. A history of congestive heart failure was seen in 5% of patients, while 5% had prior stroke/TIA and 5% had stage ≥ 3b CKD. Of the 60 (10%) patients with Af, 25 had paroxysmal, 10 had persistent, and 25 had permanent type of Af. The QRS cut-off of 110 ms was based on the upper limit of the normal range for the Japanese population. Of the 16 patients with a QRS duration ≥ 120 ms, 14 had complete right bundle branch block (CRBBB), including 1 patient with Wolff-Parkinson-White syndrome. Among CNS agents, opioids were used only by 2 patients, both for pain relief from orthopedic diseases (Supplemental Table).

### Sleep variables detected by CPAP remote monitoring

Sleep variables derived from the CPAP device are shown in Table 2. The average CPAP use time was 381.0 ± 74.4 min, and the average percentage of days of CPAP use was 95.5 ± 9.4%, indicating excellent adherence to CPAP treatment. The median AHI of 3.1 times/h also indicates favorable control of SAS in almost all patients. The median PB%Device in stable OSA patients on CPAP was low at 0.32% with a 75th percentile of 0.94%. The average log PB%Device was − 0.43 ± 0.64, which corresponds to a PB%Device of 0.37% ± 2.360 (0–32.8%). Thus, of the 618 patients, 149 (24%) had PB% of ≥ 1%.

### Relationship between indexes of apnea event and log PB%

**Fig. 2:**
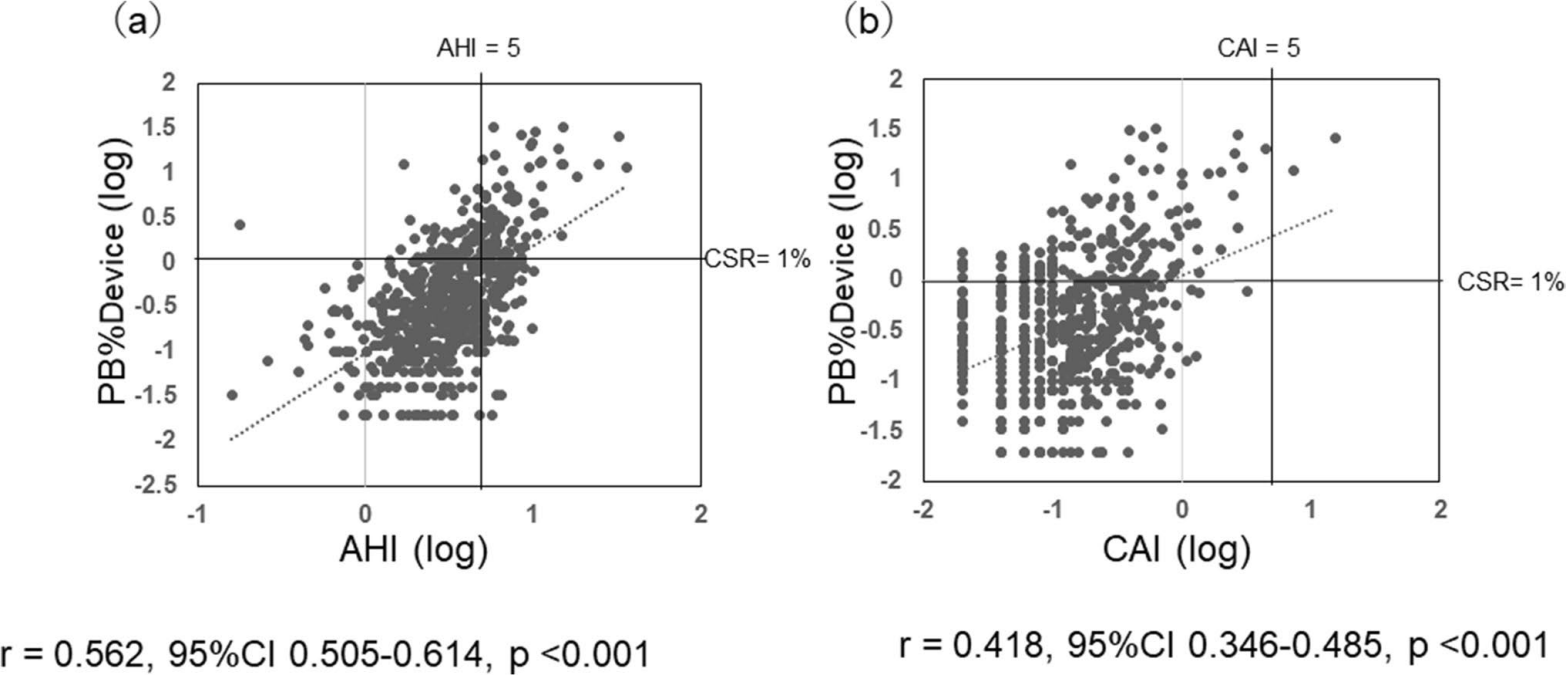
Relationship between indexes of apnea event and log PB% from the CPAP devices. PB%Device are plotted against their log AHI (**a**) and log CAI (**b**). Note the significant correlation between log PB%Device and log AHI (*r* = 0.562, *p* < 0.001), log CAI (*r* = 0.418, *p* < 0.001). *PB*, periodic breathing; *CPAP*, continuous positive airway pressure; *AHI*, apnea/hypopnea index; *CAI*, central/clear apnea index

Figure 2a and b show the correlations between log AHI and log PB%Device and between log CAI and log PB%Device, respectively. Patients with AHI ≥ 5 with poorly controlled OSA on CPAP tended to have higher PB%Device values and showed a moderate correlation between AHI and PB%Device with *r* = 0.562 (95% CI: 0.51–0.61). Of the 466 patients whose SAS was well controlled with AHI < 5, 66 (14.2%) had PB%Device ≥ 1%, with the minimum being 1% and the maximum being 14.3%. The correlation between log CAI and log PB%Device was moderate with *r* = 0.42, and only 2 patients had CAI ≥ 5.

### Multiple linear regression analysis for log PB%Device (*n* = 607)

The results of linear multiple regression analysis of factors associated with PB (log PB%Device) are shown in Table 3. Eleven patients had a PB% of 0, which was considered as missing data. The *p* value of *F*-test was < 0.001, indicating that the current model was effective. The most important factor was atrial fibrillation (Af) with a coefficient of 0.693 (95% CI; 0.536 to 0.851), followed by QRS duration coefficient of 0.445 (95% CI; 0.304 to 0.586), followed by history of heart failure, male, comorbid hypertension, obesity, and age. Contrastingly, CKD, prior stroke/TIA, and CNS agents were not strongly associated with CSB onset. Finally, the following regression equation was obtained.

**Table 3:** Multiple linear regression analysis for Log PB%Device (n = 607)

Factor variables	Coefficient (95%CI)	SE	t value	P value	vif
(Intercept)	-1.464 (-1.766 to -1.161)	0.154	-9.502	** < 0.001**	
Age	**0.011 (0.006 to 0.015)**	0.002	5.008	** < 0.001**	1.345
Male	**0.213 (0.057 to 0.368)**	0.079	2.680	**0.008**	1.231
BMI ≥ 30	**0.136 (0.019 to 0.254)**	0.060	2.276	**0.023**	1.233
Alcohol consumption	0.010 (-0.083 to 0.103)	0.047	0.213	0.831	1.091
Smoking status	-0.046 (-0.140 to 0.048)	0.048	-0.953	0.341	1.140
Atrial fibrillation	**0.693 (0.536 to 0.851)**	0.08	8.658	** < 0.001**	1.170
History of CHF	**0.227 (0.031 to 0.423)**	0.100	2.274	**0.023**	1.460
Hypertension	**0.143 (0.049 to 0.237)**	0.048	2.995	**0.003**	1.174
Prior stroke/TIA	0.029 (-0.173 to 0.232)	0.103	0.285	0.776	1.058
CKD above stage3b	-0.011 (-0.223 to 0.202)	0.108	-0.097	0.923	1.057
History of IHD	-0.142 (-0.327 to 0.043)	0.094	-1.503	0.133	1.249
Pacemaker implanted	-0.28 (-0.721 to0.161)	0.225	-1.246	0.213	1.178
QRS duration ≥ 110	**0.445 (0.304 to 0.586)**	0.072	6.213	** < 0.001**	1.073
central nervous system agents	-0.143 (-0.310 to 0.024)	0.085	-1.680	0.093	1.019
Large Leak ≥ 15%	0.120 (-0.074 to 0.313)	0.098	1.216	0.224	1.050

Regression coefficient values are presented with their 95% confidence intervals (CI).

* *p* values ≤ 0.05 were considered significant. (statistical significance is indicated in bold)

Adjust R = 0.536 Adjusted R^2^ = 0.287 F 15,591 = 17,27 *p* < 0.001 PB*,* periodic breathing; *SE,* standard error; *CI,* confidence interval; *Vif,* variance inflation factor; *BM*I, body mass index;

*Alcohol consumption,* 3 or more times per week; *Smoking status,* former or current smoker; *CHF,* congestive heart failureheart failure; *TIA,* transient ischemic attack; *CKD,* chronic kidney disease; *IHD,* ischemic heart disease

11 cases were missing PB%Device at 0%.

Equation for multiple regression:[Log PB%Device = -1.464 + 0.011 × age + 0.213 × male (1) + 0.136 × BMI ≥ 30 (1) + 0.693 × Atrial fibrillation (1) + 0.227 × History of CHF (1) + 0.143 × Hypertension (1) + 0.445 × QRS duration ≥ 110 (1)]

Equation for multiple regression: [log PB%Device =  − 1.464 + 0.011 × age + 0.213 × male (1) + 0.136 × BMI ≥ 30 (1) + 0.693 × Atrial fibrillation (1) + 0.227 × History of CHF (1) + 0.143 × Hypertension (1) + 0.445 × QRS duration ≥ 110 (1)].

### Comparison of log PB% and log AHI from the CPAP devices: paroxysmal Af group vs. persistent Af group vs. permanent Af group

**Fig. 3:**
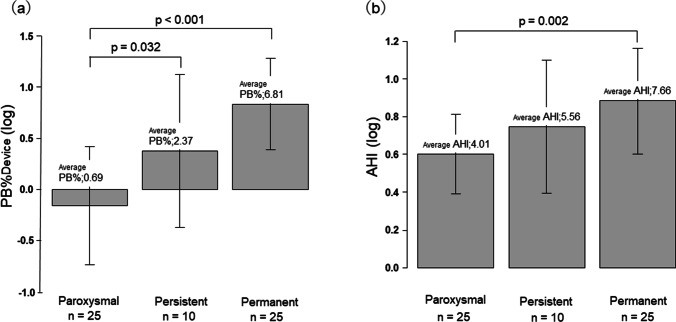
Comparison of log PB% and log AHI from the CPAP devices: paroxysmal Af group vs. persistent Af group vs. permanent Af group. Average PB%Device, 0.69 vs. 2.37 vs. 6.81, respectively. Log PB%Device was significantly higher in the persistent Af group and permanent Af group than in the paroxysmal Af group (*p* = 0.032, *p* < 0.001). Average AHI, 4.01 vs. 5.56 vs. 7.66, respectively. Log AHI was significantly higher in the persistent Af group than in the paroxysmal Af group (*p* = 0.002). *p ≤ 0.05, one-way ANOVA followed by Tukey’s post hoc test. *PB*, periodic breathing; *AHI*, apnea/hypopnea index

In the comparison of log PB%Device by type of Af, no significant difference was observed between permanent (average PB% = 6.81, *n* = 25) and persistent (average PB % = 2.37, *n* = 10) types (Fig. 3a), while there were significant differences between paroxysmal (average PB % = 0.69, *n* = 25) and persistent types (*p* = 0.032) and between paroxysmal and permanent types (*p* < 0.001). For log AHI, a significant difference between paroxysmal and permanent types (*p* = 0.002; Fig. 3b) was observed.

### Comparison of log PB% and log AHI from the CPAP devices: 120 ms > QRS duration ≥ 110 ms group vs. QRS duration ≥ 120 ms group

**Fig. 4:**
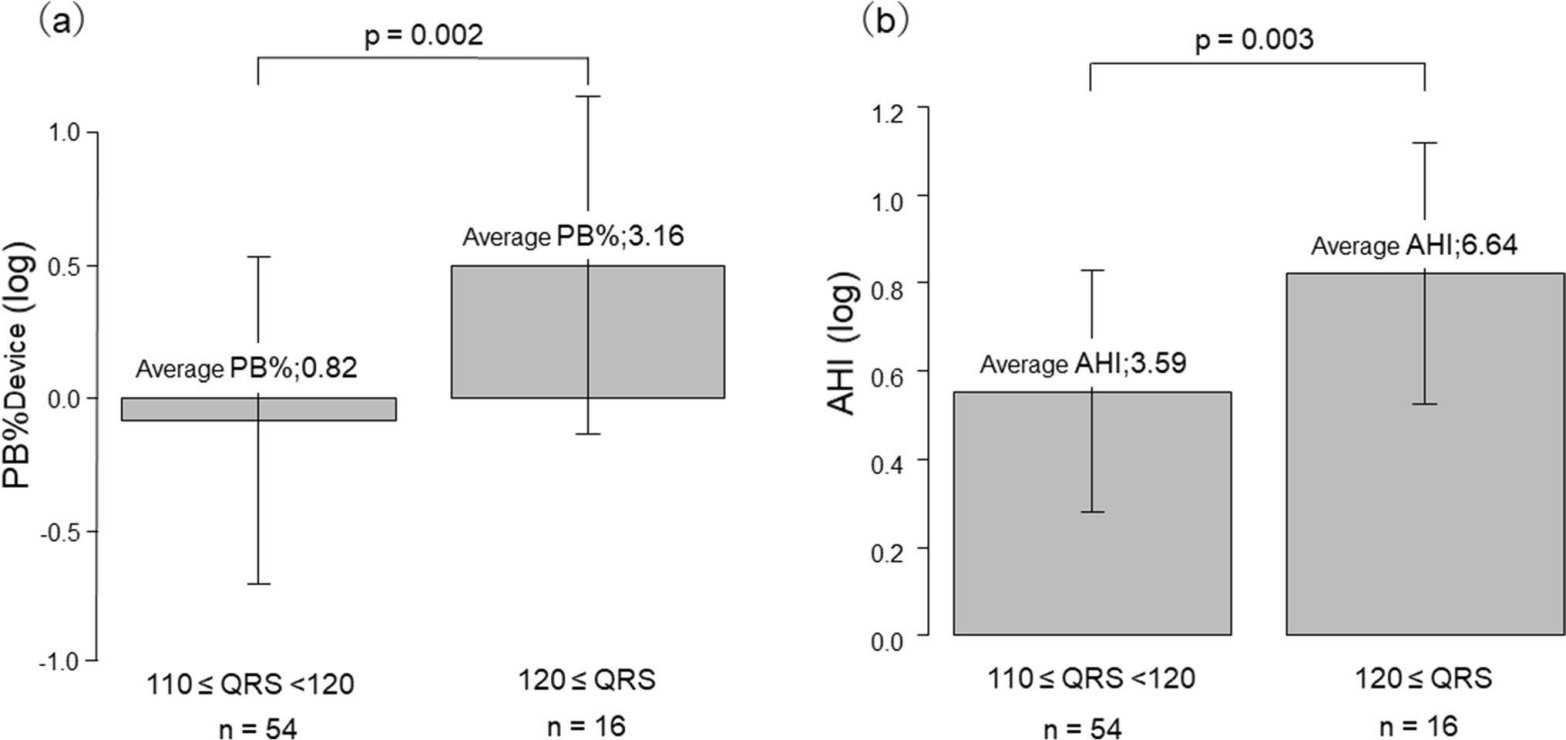
Comparison of log PB% and log AHI from the CPAP devices: 110 ≤ QRS duration < 120 group vs. 120 ≤ QRS duration group. Average PB%Device, 0.82 vs. 3.16, respectively. Log PB%Device was significantly higher in the 120 ≤ QRS duration group than in the 110 ≤ QRS duration < 120 group (*p* = 0.002). Average AHI, 3.59 vs. 6.64, respectively. Log AHI was significantly higher in the 120 ≤ QRS duration group than in the 110 ≤ QRS duration < 120 group (*p* = 0.003). *p ≤ 0.05, by Welch two-sample *t*-test. *PB*, periodic breathing; *AHI*, apnea/hypopnea index

In comparisons between subgroups based on abnormal QRS duration on ECG, patients with QRS duration ≥ 120 ms group showed significantly higher log PB%Device values (average PB%: 3.16%, *n* = 16) than those with 120 ms > QRS duration ≥ 110 ms group (average PB%: 0.82%, *n* = 54; *p* = 0.002; Fig. 4a). Similarly, log AHI values were also significantly higher in those with QRS duration ≥ 120 ms group (average AHI: 6.64, *n* = 16) than those with 120 > QRS duration ≥ 110 ms group (average AHI: 3.59, *n* = 54; *p* = 0.003; Fig. 4b).

### Investigation of factors for CSB onset in the persistence group and the emergence group

In 61 of 79 patients with PB%Device ≥ 2%, we re-examined the data from diagnostic PSG, calculated PB%PSG, and compared PB%PSG with PB%Device. Table 4 shows clinical factors of the persistence group and the emergence group.

**Table 4:** Clinical data of patients with PB%Device≧ 2% (*n* = 61)

	Persistence	Emergence
Variables	PB%PSG≧1% (n = 37)	PB%PSG < 1% (n = 24)
PB%PSG	8.1 ± 8.0	0.1 ± 0.2
PB%Device	8.9 ± 8.3	4.3 ± 3.1
Past History of Congestive heart failure	13 (35.1)	5 (20.8)
Exacerbation or new on set Congestive heart failure		**6 (25.0)**
Prior Stroke/TIA	5 (13.5)	1 (4.2)
New on set Stroke/TIA		**1 (4.2)**
Atrial fibrillation (comorbidity or relapse)	19 (51.4)	**7 (29.2)**
QRS > 110 ms	13 (35.1)	**10 (41.7)**
Echocardiogram findings		
Average E/e'	14.0 ± 4.1	12.8 ± 3.9
Average E/e' > 14	8 (21.6)	**4 (16.7)**
Ejection fraction	62.7 ± 6.9	64.3 ± 5.7
Ejection fraction ≦ 0.45	0	0
Left atrial dimension	46.1 ± 11.7	40.3 ± 7.4
Central nervous system agents	2 (5.4)	0
Alcohol consumption ≥ 3 times/week	10 (27.0)	9 (37.5)

Data are presented as n (%) or mean ± SD

*PB,* periodic breathing; *PSG,* polysomnography; *TIA,* transient ischemic attack

Echocardiogram Values missing for 14 patients with Persistence cases of PB

Echocardiogram Values missing for 12 patients with Emergence cases of PB

In the persistence group, PB%PSG was 1% or higher at baseline in 37 of 61 patients (60.7%). Of these 37 patients, 33 (89.2%) showed at least one of the following findings: past history of CHF, stroke, and atrial fibrillation; diastolic dysfunction detected by echocardiography between 2019 and 2020; and increased QRS duration detected by electrocardiography between 2019 and 2020 (CHF in 13 patients, cerebral infarction in 5 patients, atrial fibrillation in 19 patients, diastolic dysfunction in 8 patients, and increased QRS duration in 13 patients). In the remaining four patients, elevated PAP device pressure was not associated with the occurrence of PB, and the frequency of PB indicated that TECSA was unlikely. Two of these patients were public transportation drivers, and their PB% increased consistently on their days off; they had a habit of drinking a relatively large amount of alcohol on days off.

In the emergence group, PB%PSG at the time of diagnosis was < 1% in 24 patients (39.3%).

Of these 24 patients, 20 (83.3%) showed at least one of the following findings during the period from diagnostic PSG to May 31, 2020: new onset or relapse of heart failure, cerebral infarction, or atrial fibrillation, diastolic dysfunction detected by echocardiography between 2019 and 2020, and increased QRS duration detected by electrocardiography between 2019 and 2020. (New onset or relapse of heart failure in 6 patients, new onset of cerebral infarction in 1 patient, comorbid atrial fibrillation in 7 patients, diastolic dysfunction in 4 patients, and increased QRS duration in 10 patients). In the remaining four patients, elevated PAP device pressure was not associated with the occurrence of PB. Their general condition was favorable, and their lifestyles did not change. The frequency of PB was approximately 2% in 3 of these 4 patients.

## Discussion

We analyzed CPAP remote monitoring data to identify factors associated with the PB in patients with stable OSA on CPAP. No report describes PB% in stable OSA patients on long-term CPAP treatment. This is the first report to derive the reference value and prediction formula for PB%.

In this cohort of stable patients with OSA without new cardiovascular events over 6 months, the median PB% was low. Nevertheless we believe that PB% has pathophysiological significance.

The correlation between log PB%Device and log AHI was *r* = 0.56. Even patients with AHI < 5, which indicated good control of OSA by CPAP, also had PB pattern. In actual clinical practice, patients are typically evaluated primarily by the AHI. Since in some cases the rise of PB% is not reflected in the AHI, we believe that at the time of the SAS patient examination, PB% must also be taken into consideration.

The prevalence of SAS in patients with chronic heart failure is reportedly 9–50% [16, 17]. Suggested predisposing factors for CSA-CSB include male sex, age ≥ 60 years, the presence of Af, and daytime hypocapnemia [18]. In the current study involving patients with stable OSA, the most strongly predictive factors for increased PB% were the presence of Af and QRS duration (especially CRBBB), followed by a history of congestive heart failure, male, comorbidity of hypertension, obesity, and age, similar to the predisposing factors of CSB in the general population reported by Donovan et al. [19].

The persistent and permanent types of Af were associated with significantly higher values of PB% and AHI compared to the paroxysmal type. Similarly, an analysis by QRS duration also showed significantly higher values of PB% and AHI in patients with QRS duration ≥ 120 ms. In both comparisons, the difference was markedly higher for PB%Device than AHI.

This study revealed that the PB patterns exhibited by the CPAP device were consistent with previously reported patterns of CSA-CSB observed under PSG.

In comparing PB%PSG and PB%Device, comorbidity of Af, history of CHF, and previous stroke contributed to the persistence of PB%. Recurrent or new onset of CHF, stroke or Af, and diastolic dysfunction and QRS width between the time of PAP implementation and May 31, 2020, were suggested to have an impact on the secular increase in PB%.

These findings suggest that the PB detected by the device may be a useful clinical indicator.

An important pathophysiological factor for the onset of CSB is the response of various receptors involved in respiratory regulation. In patients with cardiac failure, increased left atrial pressure, pulmonary congestion, delayed circulation time, and increased sympathetic activity have been associated with CSB [20].

The association between CSB and Af has also been described [21]; Af is considered to cause CSA through a mechanism similar to congestive heart failure. Increasing pulmonary vascular resistance caused by the increased left atrial pressure stimulates the pulmonary vagal receptors, causing hyperventilation and hypopnea [22, 23].

Patients with Af, even with normal systolic left ventricular function, have been reported to have a higher prevalence of CSB [24], suggesting that CSB is more attributable to left ventricular end diastolic pressure or pulmonary congestion than to left ventricular systolic function itself.

Some authors have suggested that the presence of CSA-CSB predicts future onset of Af [25]. They state that the presence of CSA-CSB may contribute to increased sympathetic activity and autonomic imbalance, which are known predisposing factors of Af. We speculate that the presence of CSB itself indicates the presence of left atrial overload and pulmonary congestion. The left atrial overload may gradually increase over time, eventually leading to overt Af, and these are not sudden changes [26]. Therefore, we believe that time-course monitoring of PB%Device is effective in predicting the onset of Af.

Regarding the association between CSB and QRS duration, we speculate the involvement of delayed circulation time and resulting delayed transmission of information to central chemical receptors. In patients with QRS duration ≥ 120 ms, CRBBB was the most frequent cause of CSB onset. Decreased right ventricular function and right ventricular enlargement have been associated with the onset of CSB [27]. Delayed circulation time due to delayed or mismatched right ventricular wall motion may also contribute to CSB. Reduced CSB in patients undergoing CRT has also been described [28]. Among the 7 patients with a history of pacemaker implantation in the current study, some had CRT device implanted with reduced left ventricular ejection fraction, and CSA-CSB was hardly observed in these patients. Joseph et al. have concluded that prolonged QRS duration in patients with heart failure and preserved left ventricular contractility is associated with increased risk of adverse clinical outcomes, regardless of the type of conduction abnormalities responsible for QRS prolongation [29].

The current study is limited in that it was conducted retrospectively and at a single institution. It needs to be validated in a prospective multicenter study.

Second, no titration under PSG was performed before the initiation of CPAP treatment. We might have been unable to completely differentiate between TECSA and non-pathological CSA-CSB observed at sleep onset or in the rapid eye movement (REM) stage in elderly patients and others. Although there are various reports on the incidence of TECSA during the early phase of positive airway pressure (PAP) treatment, TECSA is self-limiting and resolves within 3 months in 90% or more of patients [30]. In a prospective study of patients with normal BNP levels, Westhoff et al. reported that the incidence of TECSA was as low as 0.56% [31]. We also consider that patients without heart disease can be treated to some extent, if remote monitoring is started soon after initiation of CPAP treatment to carefully observe them for the timing of pressure increase and PB onset, as well as the amount of leakage.

Third, because the data produced by the algorithm of the CPAP device were not fully validated, we were unable to detect central sleep hypopnea. However, if longitudinal monitoring of PB as a marker leads to early detection of heart disease, the algorithm may have great clinical benefit. In the future, the accuracy of the algorithm may need to be further improved through collaboration between the fields of medicine and engineering.

In conclusion, the median PB% in stable OSA patients was low with only 24% of patients having PB% ≥ 1%. Furthermore, perpetuating factors of Af and an increase in QRS duration were identified as important predictors of increased PB%.

Conversely, increased PB%Device may predict the progression of pulmonary congestion that signals the onset and relapse of Af and heart failure.

## Supplementary Information

Below is the link to the electronic supplementary material.

Supplementary file1 (DOCX 18 KB)

Supplementary Fig. 1 (PNG 29 KB)

High Resolution Image (TIF 2809 KB)

## Data Availability

The datasets used and/or analyzed during the study are available from the corresponding author on direct request.
