# Supplementary material for: Periodic breathing in patients with stable obstructive sleep apnea on long-term continuous positive airway pressure treatment: a retrospective study using CPAP remote monitoring data
Source: Sleep Breath. 2021 Oct 14;26(3):1181–91. doi: 10.1007/s11325-021-02510-0 (PMC9418282; doi:10.1007/s11325-021-02510-0)
Supplement: Supplementary file 1 — Supplementary file1 (DOCX 18 KB) [file 11325_2021_2510_MOESM1_ESM.docx]

**Supplementary information**

Supplemantal Table

Supplemental Figure

**Figure Legends**

**Supplemental Figure** 　Titration algorithm in our clinic

*CPAP*, Continuous positive airway pressure; *TECSA*, Treatment-emergent central sleep apnea; *CHF,* Congestive heart failure; *BNP*, Brain natriuretic peptide; *ASV*, Adaptive servo-ventilation; *PSG*, Polysomnography

**Supplemental Table** Further classifications of each element

| Variables | OSA (n = 618) |
| --- | --- |
| Artrial fibrillation | 60 (9.7) |
| Paroxysmal | 25 |
| Persistent | 10 |
| Permanent | 25 |
| QRS duration ≥ 110(ms) | 70 (11.3) |
| Complete Right Bundle Branch Block | 13 |
| Incomplete right bundle branch block | 17 |
| Left bundle branch block | 1 |
| Bifascicular block | 1 |
| Wolff-Parkinson-White syndrome | 1 |
| Nonspecific intraventricular conduction delay | 37 |
| central nervous system agents | 45 (7.3) |
| opioid analgesic | 2 |
| benzodiazepine | 21 |
| non‐benzodiazepine | 20 |
| others | 8 |

Data are presented as n (%)

7 cases had concomitant use of central nervous system agents.
