# Supplementary figures and images for: Periodic breathing in patients with stable obstructive sleep apnea on long-term continuous positive airway pressure treatment: a retrospective study using CPAP remote monitoring data
Source: Sleep Breath. 2021 Oct 14;26(3):1181–91. doi: 10.1007/s11325-021-02510-0 (PMC9418282; doi:10.1007/s11325-021-02510-0)

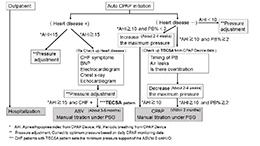

Supplement: Supplementary file 2 — (PNG 29 KB) [file 11325_2021_2510_Fig5_ESM.png]

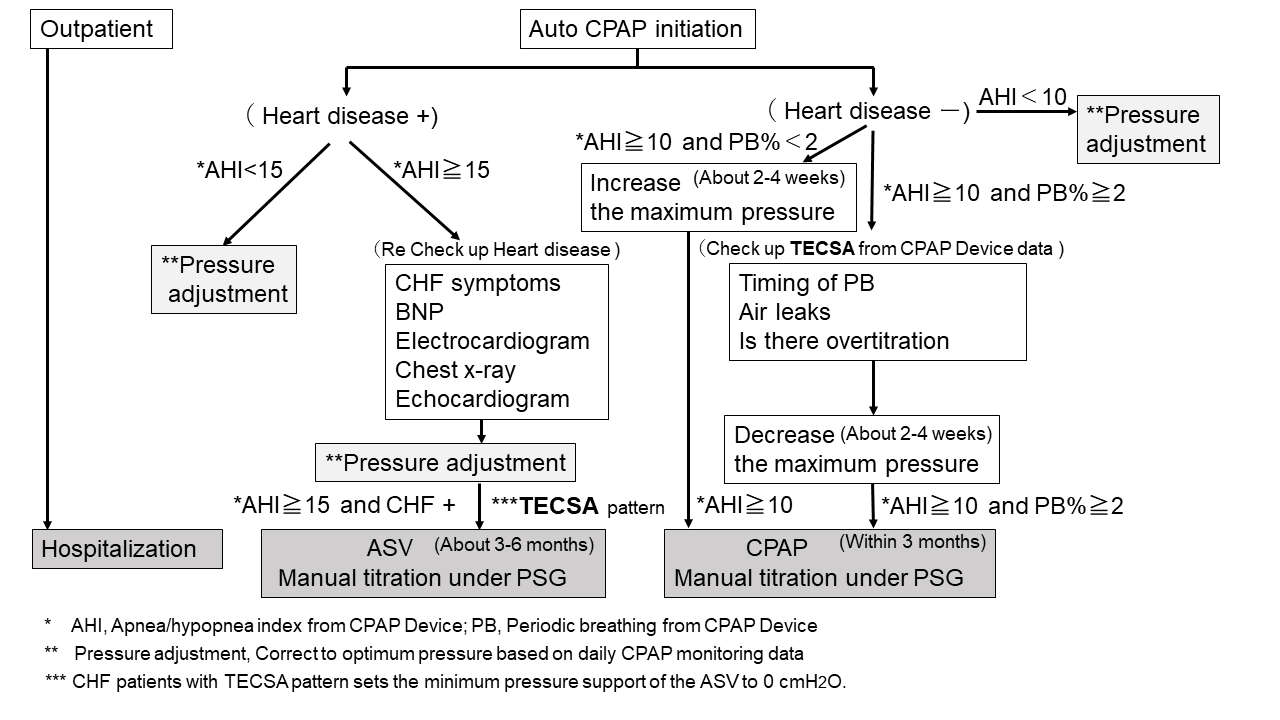

Supplement: Supplementary file 3 — High Resolution Image (TIF 2809 KB) [file 11325_2021_2510_MOESM2_ESM.tif]
